# Effect of increased dietary crude protein levels on production performance, nitrogen utilisation, blood metabolites and ruminal fermentation of Holstein bulls

**DOI:** 10.5713/ajas.18.0125

**Published:** 2018-05-31

**Authors:** Chuanqi Xia, Muhammad Aziz Ur Rahman, He Yang, Taoqi Shao, Qinghua Qiu, Huawei Su, Binghai Cao

**Affiliations:** 1 State Key Laboratory of Animal Nutrition, College of Animal Science and Technology, China Agricultural University, Beijing 100193, China; 2 Institute of Animal and Dairy Sciences, University of Agriculture Faisalabad, Faisalabad 3800, Pakistan; 3 Department of Animal Sciences, University of Illinois at Urbana-Champaign, Urbana, IL 61801, USA

**Keywords:** Crude Protein, Nutrient Intake, Nitrogen Utilisation, Ruminal Fermentation, Holstein Bull

## Abstract

**Objective:**

This study investigated the effect of dietary crude protein (CP) supplementation on nutrient intake, nitrogen (N) utilisation, blood metabolites, ruminal fermentation and growth performance of young Holstein bulls.

**Methods:**

Twenty-one young bulls weighing 277±11.2 kg were equally divided into three groups and were offered diets formulated with low CP (LCP; 10.21% CP and 4.22% rumen degradable protein [RDP]), medium CP (MCP; 12.35% CP and 5.17% RDP) and high CP (HCP; 14.24% CP and 6.03% RDP). Yellow corn silage was used as a unique forage source and was mixed with concentrate. This mixed feed was given *ad libitum* to the young bulls included in the study.

**Results:**

Results showed that CP intake, blood urea nitrogen, N intake, total N excretion and N balance increased linearly with an increase in dietary CP level (p<0.05). However, no significant difference was observed in nutrient digestibility among the bulls receiving the different diets. Ruminal pH (p<0.05) and ammonia nitrogen (NH_3_-N) concentration (p<0.01) were significantly higher in the bulls receiving the MCP and HCP diets than in those receiving the LCP diet. The bulls receiving the HCP diet showed significantly higher ruminal bacterial protein level, propionate, acetate and total volatile fatty acid (TVFA) concentrations than bulls receiving the LCP diet (p<0.05). Moreover, dietary CP level exerted a significant positive effect on the final body weight, average daily gain and gain-to-feed ratio of the bulls (p<0.05).

**Conclusion:**

High dietary CP level is optimal for achieving maximum growth and high profitability without exerting a negative effect on the physiology of growing Holstein bulls.

## INTRODUCTION

Corn straw silage is considered as a type of momentous roughage source in cattle husbandry and is widely used in China, especially in northern China, because of its accessibility and price. However, corn straw normally contains high level of fibre, but insufficient level of protein. Suharti et al [1] suggested that inadequate availability of nutrients in a high roughage-containing diet might be the major cause of low production in cattle. Moreover, dietary crude protein (CP) is crucial for promoting ruminal fermentation and nutrient digestibility. Protein supplementation of low-quality coarse diet may improve roughage utilisation and productive performance of cattle [2].

Previous studies have shown that inadequate rumen degradable protein (RDP) level reduces ammonia nitrogen (NH_3_-N) concentration in rumen fluid, which may decrease microbial growth, protein synthesis and fermentation [3]. Nevertheless, excessive protein fed to ruminants is degraded to NH_3_-N. Moreover, absorption of excessive NH_3_-N into the blood increases urea conversion in the liver, which is eventually lost through urine [4]. However, excessive nitrogen results in economic loss, adverse environmental effects and possibly some metabolic diseases [5]. Therefore, it is important to feed appropriate CP rations to cattle because of the environmental impacts from nitrogen excretion.

Many studies have adopted dietary nutrition strategies to investigate the effect of different protein levels on nutrient intake, digestion and metabolism in cattle. Nutrient intake and apparent digestibility increase with an increase in dietary CP level [6]. Some literatures have shown that an increase in dietary protein level significantly increases blood urea-nitrogen (BUN) level [7], ruminal NH_3_-N concentration [8,9] and urinary N excretion [10] and decreases N utilisation efficiency [11]. Ruminal fermentation can be improved because cattle receiving diets containing high CP level show significantly higher bacterial population [6], microbial protein synthesis [7] and volatile fatty acid (VFA) concentration in rumen fluid [9,12] than cattle receiving diets containing low CP level. Previous studies evaluating optimal dietary CP levels for cattle have provided varied results [11,13]. However, most of these studies have focused on mature dairy cows and finishing cattle. Moreover, studies assessing the effects of dietary protein levels on young Holstein bulls (age, 9 to 12 months) are limited. Young animals aged 9 to 12 months show efficient body tissue deposition and have high nutrition requirement, particularly high protein requirement [14]. Galyean [15] suggested that dietary CP should be between 12.5% and 14.4% of dry matter (DM) basis for beef cattle. Based on NRC recommendations and Galyean [15] study, one can confuse the dietary inclusion of CP in the diet of beef cattle. We used NRC [14] recommendations level of CP (12.5%) as reference in the diet of growing bulls and compared with Galyean [15] recommendations. Therefore, with reference diet, two additional CP levels (low and high) diets were also formulated.

In China, feeding system involving the specific utilisation of yellow-corn straw diet for rearing cattle is inferior. Therefore, the present study investigated the effect of dietary CP levels on feed intake, apparent nutrient digestibility, N metabolism, ruminal fermentation, bacterial protein and growth performance of Holstein bulls. Moreover, this study determined the optimal dietary CP level for improving N balance in young bulls without compromising nutrient digestibility and growth performance. Finally, this study provided a theoretical basis for formulating diets and optimising protein feed utilisation in Holstein bulls during the growing period.

## MATERIALS AND METHODS

This study was conducted in the Zhuozhou City of Hebei Province of China (latitude: 39°21′N, longitude: 115°44′E and altitude: 50 m). Experimental bulls included in this study were cared for according to the practices mentioned in the Guide for the Care and Use of Animals in Agricultural Research and Teaching of the Animal Care and Use Committee, China Agricultural University (Beijing, China).

### Animals and feeding management

The study included 21 Chinese Holstein bulls with an average age of nine months and average initial body weight (BW) of 277±11.2 kg (mean±standard deviation). The bulls were randomly allocated to receive the following three dietary treatments (seven animals per treatment) for an experimental period of three months: low crude protein (LCP; 10.21% CP and 4.22% RDP), medium crude protein (MCP; 12.35% CP and 5.17% RDP) and high crude protein (HCP; 14.24% CP and 6.03% RDP) on DM basis were formulated referring to NRC [14] and Galyean [15] recommendations. The study was divided into two periods, namely, a 15-day adaptation period and three-month experimental period. In the present study, yellow corn silage was used as a unique forage source and was mixed with concentrate; moisture content of the diet was adjusted to approximately 55% of the DM. The mixed feedstuff was fed to the experimental young bulls *ad libitum* (5% refusal was permitted). The animals were located in individual stalls and were fed twice daily at 0800 and 1800 h. Moreover, bulls in all the experimental groups were provided water *ad libitum*. The ingredients and chemical composition of the experimental diets are presented in Table 1.

### Data collection and sampling

The feed given and residuals were collected and recorded each day to calculate average dry matter intake (DMI). Weekly residual, yellow corn silage and concentrate were gathered from approximately 500 g samples and were stored at −20°C. The animals were individually weighed before the morning feeding at the start and end of the experiment to calculate weight gain; moreover, body size was measured simultaneously. Wither height (distance from the base of the front feet to the withers), body length (distance between the points of the rump and shoulder), heart girth (circumference of the chest), body barrel (circumference of the belly) and waist (circumference of the waist) were measured according to a procedure described in our previous study [16]. Body size increments were calculated based on differences in body size measurements of the bulls at the start and end of the experiment. Average daily gain (ADG) and gain-to-feed ratio (G:F) were calculated by dividing BW gain by the number of experimental days and total DMI, respectively.

#### Blood sampling

On the last day of each month, blood samples (approximately 10 mL) were collected from the jugular vein of each bull before the morning feeding in tubes containing sodium heparin as an anticoagulant. Plasma samples were separated by centrifuging at 1,000×g for 20 min, and supernatant obtained was stored at −20°C for further analysis. Glucose, total cholesterol, triglyceride, BUN and total protein concentrations were determined using applicable kits (Jiuqiang Biological Technology Co., Ltd, Beijing, China) and an automated analyser (HITACHI 7020 Automated Biochemical Analyzer; HITACHI, Tokyo, Japan).

#### Ruminal fluid sampling

At the end of the experiment, ruminal fluid sample was collected from each bull at 0, 2, 4, 6, 8, and 10 h after the morning feeding by using a flexible oral stomach tube. Ruminal samples obtained over three consecutive days (d 1st at 0800 and 1400 h, d 2nd at 1000 and 1600 h, d 3rd at 1200 and 1800 h) were combined to represent a single feeding phase. The samples were filtered using four layers of cheesecloth, and ruminal pH was measured immediately by using a pH meter (PHS-3C; Leici Scientific Instrument Co., Ltd., Shanghai, China). Next, the filtrate was centrifuged at 10,000×*g* for 15 min. Approximately 10 mL supernatant was acidified using 3 mL 25% metaphosphoric acid solution and was frozen at −20°C prior to further analysis. Ammonia nitrogen concentration was measured using a spectrophotometer (UV-1700; Shimazu Corp., Kyoto, Japan) according to a method described by Broderick and Kang [17]. The VFA concentration was analysed by performing gas chromatography (GC-2014; Shimazu Corp., Japan) with a capillary column (RTX-WAX, 30 m×0.25 mm×0.25 μm; Shimazu Corp., Japan), according to a method described by Kim et al [18]. Gas chromatography was performed using an automatic injector with a split ratio of 50:1 and a temperature of 220°C. Injection volume was maintained at 0.4 μL, air flow was maintained at 450 mL/min, hydrogen flow was maintained at 40 mL/min and nitrogen flow was maintained at 45 mL/min. The detector temperature was set at 250°C. Individual VFAs were identified by comparing relative retention times with those of standard mixtures.

Residual rumen liquid was used for determining bacterial protein level by performing differential velocity centrifugation according to a method described by Berger et al [19]. Briefly, the rumen liquid was centrifuged at 500×*g* for 20 min to remove solid feedstuff particles and protozoa. Supernatant obtained was fixed with 0.9% sodium chloride-formalin solution (supernatant:solution ratio = 4:3), and total mixed liquid (approximately 225 mL) was centrifuged at 22,000×*g* for 20 min. Next, sediment was dried in an oven for 3 h for obtaining the rumen liquid-associated bacterial protein.

#### Faeces and urine sampling

In the final three consecutive days of the experiment, the animals were tethered to determine the total-tract apparent digestibility of nutrients by using a total collection method. Faecal was thoroughly mixed and approximately 200 g sample (fresh weight) was collected. The samples (5% fresh weight) were then mixed with tartaric acid (10% solution), dried in an oven (65°C for 72 h), then ground through a 1-mm sieve and stored for chemical analysis. Digestibility was calculated using nutrient concentration in the faeces and the diet consumed. Total daily urine was collected from each bull during the final three days of the experiment by using a plastic barrel connected to a pipe and a funnelled rubber bag fixed to the hypogastrium (covering the urethral opening of the bull). The urine was acidified using 3.6 mol/L sulfuric acid solution to maintained its pH at <3.0 Next, approximately 50 mL acidified urine was sampled and stored at −20°C for analysing N content. Feed N balance was calculated by subtracting mean N excretion (faecal and urinary N excretion) from the mean N intake.

### Chemical analysis

Chemical analyses of the yellow corn silage, concentrate and faecal samples were performed for determining DM (method 930.15) and crude ash (method 942.05) content, according to methods described by the Association of Official Analytical Chemistry (AOAC) [20]. Moreover, calcium (method 927.02) and phosphorus (method 965.17) concentrations in each ashed sample were determined using methods described by the AOAC [20]. Organic matter (OM) concentration was calculated as the difference between DM and crude ash concentrations. Crude protein was calculated using the formula 6.25×N, which was determined using Kjeldahl method (method 984.13) [20]. Acid detergent fibre (ADF) (method 973.18) [20] and neutral detergent fibre (NDF) with heat-stable α-amylase were measured in accordance with methods described by Van Soest et al [21].

### Statistical analysis

Data were analysed using the PROC general linear model in SAS software (SAS Inst. Inc., Cary, NC, USA). The model used treatment (effect of diet), animals and overall error as the sources of variation. All the variables were considered to be fixed, except the animal and overall error, which were considered to be random. The following formula was used for performing data analysis:

$${\text{Y}}_{\text{ij}}=\mu +{\text{P}}_{\text{i}}+{\text{A}}_{\text{j}}+{\text{e}}_{\text{ij}},$$

Where Y_ij_ = each observation, μ = overall mean, P_i_ = fixed effect of dietary protein level, A_j_ = random effect of animal and e_ij_ = error term.

Results for ruminal metabolism were analysed using PROC MIXED function available in the SAS software. The model used for ruminal parameters (pH, NH_3_-N, and VFA concentrations and bacterial protein level), which included repeated measurements taken from the same animal, used treatment (effect of diets), animals, hours postfeeding (repeated measures being hours for ruminal fluid), treatment×hours postfeeding interaction and overall error as the sources of variation. All the variables were considered to be fixed, except the animal variable, which was considered to be random. Data analysis was performed using the following formula:

$${\text{Y}}_{\text{ijk}}=\mu +{\text{P}}_{\text{i}}+{\text{T}}_{\text{j}}+{\left(\text{P}\times \text{T}\right)}_{\text{ij}}+{\text{A}}_{\text{k}}+{\text{e}}_{\text{ijk}}$$

Where Y_ijk_ = each observation, μ = overall mean, P_i_ = fixed effect of dietary protein level, T_j_ = fixed effect of hours postfeeding, (P×T)_ij_ = fixed effect of treatment×hours postfeeding interaction, A_k_ = random effect of animal and e_ijk_ = error term.

Effect of the diets on response variables was analysed using orthogonal contrasts (linear and quadratic). A probability value of p<0.01 indicated an extreme significant difference, p<0.05 indicated a significant difference and p<0.10 indicated a tendency. Correlation coefficients among the variables were determined using Pearson correlation analysis, and Heatmap analysis was conducted using R V.3.2.3 software.

## RESULTS

### Feed intake and apparent total-tract nutrient digestibility

Effects of different dietary CP levels on feed intake and apparent total-tract nutrient digestibility are presented in Table 2. Dietary CP level significantly affected the CP intake by bulls (p<0.01). The bulls receiving the HCP and MCP diets showed higher CP intake than those receiving the LCP diet (0.93, 0.86, vs 0.77 kg/d, respectively). However, DM, ADF (p = 0.057), and NDF (p = 0.086) intake was similar among the bulls receiving the different diets. In addition, no significant difference was observed in the apparent total-tract digestibility of each nutrient among the bulls receiving the different diets.

### N metabolism

Crude protein supplementation in the diet had a positive linear effect on N intake, total N excretion and N balance (p<0.05) (Table 3). The bulls receiving the HCP and MCP diets showed higher N intake than those receiving the LCP diet (148.35, 138.09 vs 123.47 g/d, p<0.01). Total N excretion and N balance were higher in the bulls receiving the HCP diet than in those receiving LCP diet (102.32 vs 94.00 g/d, p<0.05; 46.02 vs 29.47 g/d, p<0.01, respectively). However, no difference was observed in total N excretion and N balance between the bulls receiving the HCP and MCP diets. Furthermore, dietary CP level had no effect on faecal N (p = 0.059) and urinary N excretion.

### Blood metabolites

The effect of dietary protein level on blood metabolites in the young bulls is shown in Table 4. Blood urea nitrogen concentration increased linearly from 2.47 to 2.95 mmol/L with an increase in dietary CP level (p<0.05). However, the concentrations of other blood metabolites were not affected linearly or quadratically by the diets containing different CP levels.

### Ruminal fermentation

**Figure 1:**
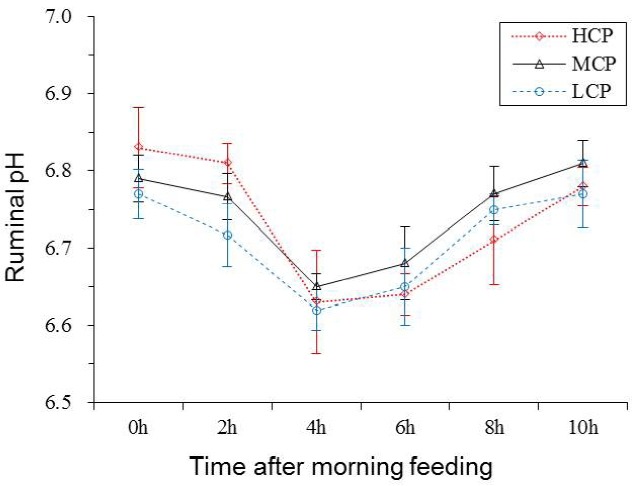
Ruminal pH value for each treatment at different sampling times after feeding. LCP, low crude protein; MCP, medium crude protein; HCP, high crude protein.

**Figure 2:**
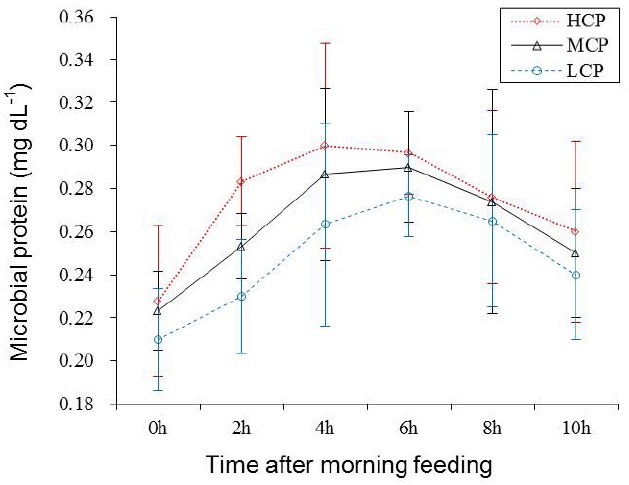
Ruminal bacterial protein level for each treatment at different sampling times after feeding. LCP, low crude protein; MCP, medium crude protein; HCP, high crude protein.

**Figure 3:**
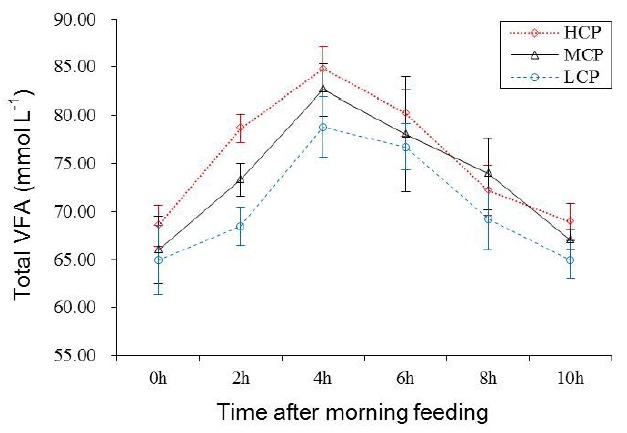
Ruminal total VFA concentration for each treatment at different sampling times after feeding. LCP, low crude protein; MCP, medium crude protein; HCP, high crude protein; VFA, volatile fatty acid.

Ruminal pH and NH_3_-N concentration of the bulls receiving the HCP and MCP diets were significantly higher than those of the bulls receiving the LCP diet (6.81, 6.77 vs 6.72 mg/dL, p<0.05; 7.29, 6.43 vs 5.00 mg/dL, p<0.01; Table 5 and Figure 1). Furthermore, the bulls receiving the HCP diet showed higher ruminal bacterial protein concentration than those receiving the LCP diet (0.28 vs 0.23 mg/dL, p<0.05). However, no difference was observed in ruminal bacterial protein concentration between bulls receiving the MCP and LCP diets (Table 5, Figure 2). In addition, the concentrations of VFAs, namely, propionate (p<0.05), acetate (p<0.01) and total VFA (p<0.01) in the rumen were significantly different among the bulls receiving the different diets. Compared with the bulls receiving the LCP diet, the bulls receiving the HCP diet showed higher concentrations of acetate (55.58 vs 49.71 mmol/L) and propionate (12.80 vs 10.13 mmol/L). Total VFA concentration in the rumen fluid increased linearly with an increase in dietary CP level (68.42, 73.29 vs 78.65 mmol/L; Table 5, Figure 3). However, ruminal acetate:propionate ratio was not significantly different among the bulls receiving the different diets.

### Growth performance

The growth performance of the bulls in each experimental group is presented in Table 6. Variations in the dietary protein levels significantly affected the final BW, ADG, and G:F (p< 0.05). The final BW, ADG, and G:F were significantly higher in the bulls receiving the HCP diet than in those receiving the LCP diet (340.0 vs 324.5 kg; 0.68 vs 0.55 kg/d; 0.091 vs 0.077, respectively, p<0.05). However, dietary CP levels did not affect the body size measurements, including body length, heart girth, body barrel and waist (p>0.05), but significantly affected wither height (p<0.05, Table 6). Furthermore, body size increments in wither height and waist of the bulls increased significantly with an increase in dietary protein levels (p<0.05); however, other body size increments in body length, heart girth and body barrel were not significant (Table 6).

### Correlation analysis

**Figure 4:**
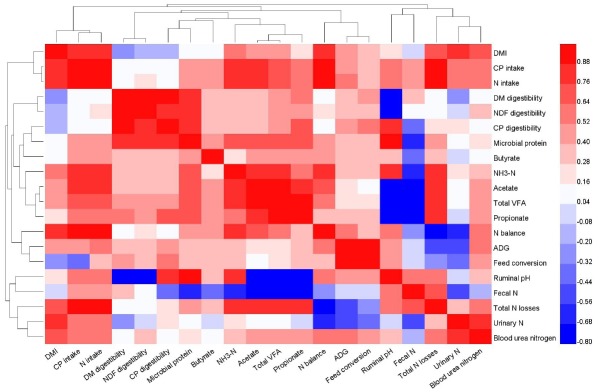
Heatmap matrix of the Pearson’s correlation analysis showing the correlation coefficients among the evaluated measurements. ADG, average daily gain; CP, crude protein; DM, dry matter; DMI, dry matter intake; N, nitrogen; NDF, neutral detergent fibre; NH_3_-N, ammonia nitrogen; VFA, volatile fatty acid.

The correlation coefficients of the evaluated measurements are presented in Heatmap Figure 4. Correlation analysis showed that ruminal pH was positively correlated with CP digestibility (r = 0.78, p<0.05) but was negatively correlated with DM digestibility (r = −0.80, p<0.05) and total VFA concentration (r = −0.76, p<0.05). A strongly negative correlation was observed between bacterial protein level and DM digestibility (r = 0.82, p<0.01) and CP digestibility (r = 0.80, p<0.01). In contrast, NH_3_-N and total VFA concentrations were positively correlated with CP intake (r = 0.84, p<0.01 and r = 0.74, p<0.05, respectively). With respect to N metabolism, a positive correlation was observed between total N loss and CP intake (r = 0.90, p<0.01) and NH_3_-N concentration (r = 0.82, p<0.01). Furthermore, N balance was positively correlated with CP intake (r = 0.97, p<0.01) and NH_3_-N concentration (r = 0.79, p<0.05), but was negatively correlated with total N loss (r = −0.77, p<0.05). Results of other correlation analyses are shown in Figure 4.

## DISCUSSION

The results of the present study indicate that CP intake increases linearly with an increase in dietary CP level, which is consistent with the result of Bahrami-Yekdangi et al [22], who also reported an improved CP intake in cows receiving a concentrate supplemented with increased CP levels. However, different dietary CP levels did not affect other nutrient parameters such as DM, NDF, and ADF intake. Previous studies have reported different results for the effect of different dietary CP levels on the apparent total-tract nutrient digestibility because of the differences in experimental conditions. In the present study, different dietary CP levels had no effect on the apparent total-tract digestibility of nutrients (Table 2). Bahrami-Yekdangi et al [23] showed that different dietary protein levels affected the total-tract digestibility of DM, OM, ADF, and NDF. However, Dung et al [24] showed that increased CP levels (10%, 13%, 16%, and 19%) resulted in an increase in CP digestibility but did not significantly affect DM, OM, and NDF digestibility, which was inconsistent with that observed in the presentstudy. Nutrient digestibility may reflect microbial activity and fermentation in the rumen; therefore, it is crucial to formulate a proper diet to increase feed digestibility and utilisation [25]. However, the insignificant difference of nutrient digestibility among different groups might due to the lesser variation of dietary CP (especially RDP) in the present study. Other possible reason of non-significant difference on nutrients digestibility could be increasing tendency of NDF and ADF intake (mainly from yellow-corn straw), which reduce the digestibility improvement by higher CP diet.

Dietary protein is degraded into ruminal NH_3_-N; however, excess NH_3_-N is converted into urinary and faecal N and is eventually secreted out of the body by ruminants. The present study showed a high correlation between N excretion and CP intake and between NH_3_-N concentration and CP intake (r = 0.90, p<0.01 and r = 0.82, p<0.01, respectively; Figure 4). The amount of excreted N reflects protein utilisation and N deposition efficiency. Bahrami-Yekdangi et al [23] found that faecal N excretion was not affected in dairy cows fed different dietary protein levels (14.8%, 15.6%, and 16.4% CP based on DM). Moreover, Reid et al [26] reported that dietary CP levels did not affect faecal and urinary N excreted by cows. The results of these studies are consistent with those of the present study, which is expected because N intake negligibly affects faecal N excretion. However, Davidson et al [27] reported that cows fed a low-protein (16.8%) diet showed significantly lower urinary and faecal N excretion compared with those fed a high-protein (19.4%) diet. Leonardi et al [10] also showed that increased dietary protein level increased urinary N excretion. However, urinary N excretion was not significantly different among the bulls receiving the different diets in the present study.

The highest N intake was observed in the bulls receiving the HCP diet. Increase in dietary CP level linearly decreased the proportion of total N excretion in N intake (76.17%, 71.88%, and 69.10% in the bulls receiving the LCP, MCP, and HCP diets, respectively). Furthermore, dietary CP level exerted a significant linear effect on N balance (Table 3). These results are consistent with the correlation coefficients between N deposition and CP intake (r = 0.97, p<0.01; Figure 4) and between N deposition and total N excretion (r = −0.77, p<0.05; Figure 4). Danes et al [11] reported that low dietary CP level increased N utilisation efficiency. In cows fed total mixed ration containing 14.8%, 15.6%, and 16.4% CP, N utilisation efficiency decreased with an increase in dietary CP level [23]. Although the bulls that were fed LCP diet showed high N efficiency; however, the bulls in HCP diet treatment showed high N deposition in present study. Therefore, it is important to provide an appropriate dietary CP concentration to young bulls in the growing stage to reduce N excretion and to improve N utilisation efficiency.

In the present study, BUN concentration increased linearly with an increase in dietary CP level. Reid et al [26] reported that grass-fed dairy cows receiving supplementary concentrates with a high CP level (302 g/kg DM) showed significantly higher BUN concentration than those receiving supplementary concentrates with a low CP level (101 g/kg DM); however, other blood variables such as glucose, total protein and albumin concentrations were not different among the cows receiving the different diets. Similarly, Gleghorn et al [13] and Pilachai et al [28] found that BUN concentration increased significantly in animals fed high-protein rations. These findings are consistent with those of the present study. The present study showed that the dietary CP level exerted a significant positive effect on ruminal NH_3_-N concentration, which may ultimately influence BUN concentration. Bahrami-Yekdangi et al [22] also noted that Holstein dairy cows receiving a high CP diet showed higher CP intake and BUN concentration.

Ruminal pH may reflect feed fermentation and internal environment in the rumen because it is influenced by nutrition levels. Increased protein content in the diet may increase NH_3_-N concentration in the rumen liquid, which may be the main reason for the increased ruminal pH value. In the present study, the results of the correlation analysis showed that ruminal pH was positively correlated with NH_3_-N concentration (r = 0.80, p<0.01; Figure 4), indicating that ruminal pH increases in animals with high NH_3_-N concentration. Ruminal pH is partly regulated by NH_3_-N concentration because consumed CP and urea are hydrolysed by microbial ureases to CO_2_ and NH_3_ in the rumen, which may lead to variations in ruminal pH [6]. However, O’Colmenero and Broderick [4] showed that ruminal pH was not affected by dietary protein levels, which was similar to that reported by Bahrami-Yekdangi et al [23] and Pilachai et al [28]. These differences may be because of VFA absorption rate, feedstuff formula variation and different dietary protein gradients in each experiment. High NH_3_-N concentration may result in the waste of N source, whereas low NH_3_-N concentration may negatively affect rumen energy and N balance and reduce rumen microbial activity, leading to a decrease in microbial protein synthesis [29]. The present study showed that the correlation coefficient between CP intake and ruminal NH_3_-N concentration was significantly high (r = 0.84, p<0.01; Figure 4). The results of the present study for ruminal NH_3_-N concentration are consistent with those of majority of previous studies. Danes et al [11] reported that dairy cows fed a high-protein (18.1%) concentrate diet showed higher ruminal NH_3_-N concentration than those fed diets containing 8.7% and 13.4% CP. Javaid et al [8] reported that increased RDP level increased ruminal ammonia/NH_3_-N concentration, indicating a positive correlation between ruminal NH_3_-N and dietary RDP level.

The results of the present study showed that dietary protein levels significantly affected ruminal bacterial protein level. The bulls receiving the HCP diet showed higher ruminal bacterial protein level than those receiving the other diets. Young bulls may more effectively utilise dietary N for growth. Vigorous metabolic activity in the rumen could serve as source of large amounts of N and energy for microbes. This was confirmed by the correlation coefficient between bacterial protein and ruminal NH_3_-N and VFA concentrations (r = 0.76, p<0.05 and r = 0.77, p<0.05, respectively; Figure 4). Bacterial population increases significantly with an increase in dietary protein levels [25]. Although dietary CP or RDP levels may significantly affect ruminal NH_3_-N concentration, they do not affect microbial protein quantity [11]. These inconsistent results of the present study might be because of the different quantity and ratio between available energy (mainly carbohydrate fermentation) and proteins used.

The VFA is the main fermentation product in the rumen, and dietary carbohydrate is the primary energy source for ruminants. The VFA concentration in the rumen is an important indicator of rumen microbial activity. Several studies have examined the effect of dietary CP levels on ruminal fermentation; however, these studies have provided inconsistent results. Pilachai et al [28] showed that VFA concentration was higher in animals fed a diet containing increased RDP level. Hatfield et al [12] reported that dietary protein level is the main factor influencing VFA concentration because acetate, propionate, butyrate, valerate, iso-butyrate, iso-valerate, and total VFA concentrations in experimental sheep fed 18% protein diet were significantly higher than those in the sheep fed 10% protein diet; however, the acetate:propionate ratio was not significantly different in the sheep fed the different diets. The results of the present study suggest that increased dietary CP level promotes ruminal fermentation in bulls. Moreover, some correlation was observed between ruminal total VFA concentration and CP intake (r = 0.74, p<0.05). However, no correlation was observed between CP digestibility and total VFA concentration in the rumen (r = 0.47, p = 0.204; Figure 4). Irrespective of this inscrutable result, the present study showed that high dietary CP level is beneficial for ruminal fermentation under the conditions used.

The final BW, ADG, and G:F are not affected by different dietary protein levels (12.4% and 14.0%) [30]. However, the results of some studies are consistent with those of the present study. Dung et al [24] found that ADG increased significantly with an increase in dietary CP level from 10% to 19%. Gleghorn et al [13] indicated that ADG in finishing beef steers increased slightly with an increase in dietary CP level from 11.5% to 13% and was significantly affected by a CP level exceeded 13%, suggesting that suitable dietary protein level should be maintained to obtain the best growth performance. However, ADG increased linearly with the increased dietary CP level, and the HCP diet was considered to be optimal for achieving the best growth performance in young bulls in the present study. A significant difference observed in CP intake, ruminal fermentation parameters, N balance, final BW, ADG, and G:F among the bulls fed the diets containing different protein levels suggests that supplementation of diet with high CP level (14.24% CP, 6.03% RDP) during the growing period is optimal for the growth of young Holstein bulls.

## Figures and Tables

**Table 1:** Ingredients and chemical composition (%) of the experimental diets (DM basis)

Item	Dietary treatments		

	LCP	MCP	HCP
Ingredients (% of DM)			
Yellow corn silage1)	45.00	45.00	45.00
Corn grain	41.53	36.51	31.49
Soybean meal	2.15	4.73	7.32
Rapeseed meal	3.69	5.82	7.96
Wheat bran	5.70	6.01	6.30
Calcium carbonate	0.55	0.55	0.55
Dicalcium phosphate	0.28	0.28	0.28
Sodium chloride	0.55	0.55	0.55
Mineral-vitamin premix2)	0.55	0.55	0.55
Chemical composition (% of DM)			
Dry matter	90.84	90.77	91.05
Organic matter	93.31	93.27	93.30
Crude protein	10.21	12.35	14.24
Rumen degradable protein3)	4.22	5.17	6.03
Total digestible nutrients3)	64.33	64.85	64.36
Neutral detergent fibre	44.35	44.55	44.29
Acid detergent fibre	23.01	23.16	23.80
Calcium	0.50	0.52	0.51
Phosphorus	0.26	0.27	0.25

DM, dry matter; LCP, low crude protein; MCP, medium crude protein; HCP, high crude protein.

1) The chemical composition of yellow corn silage (DM basis) was 92.33% for organic matter, 73.69% for neutral detergent fibre, 43.19% for acid detergent fibre and 6.5% for crude protein.

2) Premix composition/kg: Vitamin A, 1,000,000 IU; Vitamin D_3_, 350,000 IU; Fe, 4,200 mg; Cu, 2,500 mg; Mn, 5,700 mg; Zn, 5,700 mg; I, 85 mg; Se, 85 mg; and Co, 30 mg.

3) Rumen degradable protein and total digestible nutrients were calculated according to NRC [14].

**Table 2:** Feed intake and apparent total-tract nutrient digestibility of bulls fed on experimental diets with different levels of crude protein

Parameters	Dietary treatments			SEM	p-value	
	
	LCP	MCP	HCP		Linear	Quadratic
Intake						
Dry matter (kg/d)	7.11	7.39	7.54	0.205	0.186	0.856
Organic matter (kg/d)	6.63	6.89	7.04	0.191	0.190	0.857
Crude protein (kg/d)	0.77^b^	0.86^a^	0.93^a^	0.024	0.004	0.774
Neutral detergent fibre (kg/d)	3.08	3.29	3.34	0.048	0.086	0.541
Acid detergent fibre (kg/d)	1.64	1.71	1.79	0.090	0.057	0.876
Apparent total-tract digestibility						
Dry matter (%)	46.74	50.25	51.51	3.652	0.387	0.834
Organic matter (%)	55.28	57.59	59.17	3.159	0.416	0.950
Crude protein (%)	45.50	47.62	49.71	2.579	0.293	0.972
Neutral detergent fibre (%)	45.14	47.22	50.12	3.682	0.377	0.904
Acid detergent fibre (%)	37.84	39.29	41.03	3.777	0.574	0.959

LCP, low crude protein; MCP, medium crude protein; HCP, high crude protein; SEM, standard error of mean.

a,b Within the same row with different superscripts means significant difference (p<0.05).

**Table 3:** Nitrogen metabolism of bulls fed on experimental diets with different levels of crude protein

Parameters	Dietary treatments			SEM	p-value	
	
	LCP	MCP	HCP		Linear	Quadratic
Total N						
N intake (g/d)	123.47^b^	138.09^a^	148.35^a^	3.833	0.004	0.774
Total N losses (g/d)	94.00^b^	99.22^ab^	102.32^a^	1.660	0.011	0.706
% of intake	76.17^a^	71.88^ab^	69.10^b^	1.403	0.011	0.767
Urinary N						
Urinary N (g/d)	26.82	27.01	28.04	2.494	0.746	0.886
% of total excreted	28.48	27.18	27.41	2.309	0.747	0.806
% of intake	21.68	19.51	18.82	1.295	0.166	0.697
Faecal N						
Faecal N (g/d)	67.18	72.21	74.29	2.187	0.059	0.654
% of total excreted	71.52	72.82	72.59	2.309	0.747	0.806
% of intake	54.50	52.38	50.29	2.579	0.293	0.972
N balance (g/d)1)	29.47^b^	38.86^ab^	46.02^a^	0.051	0.009	0.877

LCP, low crude protein; MCP, medium crude protein; HCP, high crude protein; SEM, standard error of mean; N, nitrogen.

1) N balance = N intake–urinary N–faecal N.

a,b Within the same row with different superscripts means significant difference (p<0.05).

**Table 4:** Blood metabolites of bulls fed on experimental diets with different levels of crude protein

Parameters	Dietary treatments			SEM	p-value	
	
	LCP	MCP	HCP		Linear	Quadratic
Glucose (mmol/L)	4.85	4.77	4.82	0.178	0.890	0.763
Cholesterol (mmol/L)	3.45	3.87	3.95	0.248	0.203	0.641
Blood urea nitrogen (mmol/L)	2.47^b^	2.64^ab^	2.95^a^	0.139	0.045	0.717
Triglycerides (mmol/L)	0.07	0.06	0.05	0.009	0.400	0.918
Total protein (g/L)	60.48	52.83	56.58	3.537	0.440	0.246

LCP, low crude protein; MCP, medium crude protein; HCP, high crude protein; SEM, standard error of mean.

a,b Within the same row with different superscripts means significant difference (p<0.05).

**Table 5:** Ruminal fermentation of bulls fed on experimental diets with different levels of crude protein

Parameters	Dietary treatments			SEM	p-value	
	
	LCP	MCP	HCP		Linear	Quadratic
Ruminal pH	6.72^b^	6.77^a^	6.81^a^	0.019	0.013	0.985
NH_3_-N (mg/dL)	5.00^b^	6.43^a^	7.29^a^	0.316	0.002	0.590
Bacterial protein (mg/dL)	0.23b	0.25^ab^	0.28^a^	0.012	0.022	0.752
Volatile fatty acids (mmol/L)						
Acetate	49.71^b^	52.42^b^	55.58^a^	3.136	0.007	0.485
Propionate	10.13^b^	11.50^ab^	12.80^a^	0.795	0.049	0.916
Iso-butyrate	0.59	0.61	0.79	0.100	0.419	0.746
Butyrate	6.41	7.19	7.83	0.516	0.142	0.459
Iso-valerate	0.75	0.71	0.73	0.044	0.237	0.659
Valerate	0.83	0.87	0.93	0.066	0.445	0.440
Total VFA (mmol/L)	68.42^c^	73.29^b^	78.65^a^	3.854	0.006	0.514
Acetate:propionate	4.91	4.56	4.37	0.410	0.871	0.380

1) LCP, low crude protein; MCP, medium crude protein; HCP, high crude protein; SEM, standard error of mean; NH_3_-N, ammonia nitrogen; VFA, volatile fatty acid.

a–c Within the same row with different superscripts means significant difference (p<0.05).

**Table 6:** Growth performance and body size measurements of bulls fed on experimental diets with different levels of crude protein

Parameters	Dietary treatments			SEM	p-value	
	
	LCP	MCP	HCP		Linear	Quadratic
Growth performance						
Final weight (kg)	324.5^b^	334.2^ab^	340.0^a^	5.059	0.047	0.819
Experimental days	91	91	91			
DMI (kg/d)	7.11	7.39	7.54	0.205	0.186	0.856
ADG (kg/d)	0.55^b^	0.61^ab^	0.68^a^	0.047	0.041	0.924
G:F (kg/kg)	0.077^b^	0.083^ab^	0.091^a^	0.003	0.033	0.903
Body size and increment						
Wither height (cm)	126.3^b^	127.7^b^	129.3^a^	0.842	0.046	0.829
Increment (cm)	4.4b	5.1^b^	6.0^a^	0.448	0.045	0.665
Body length (cm)	139.0	142.0	142.3	2.11	0.301	0.652
Increment (cm)	10.0	10.8	12.3	0.957	0.149	0.748
Heart girth (cm)	173.3	175.7	176.3	3.771	0.590	0.878
Increment (cm)	11.8	12.6	13.8	1.110	0.298	0.803
Body barrel	200.2	203.7	205.3	5.721	0.531	0.909
Increment (cm)	9.1	10.4	11.3	0.888	0.122	0.856
Waist (cm)	172.3	175.3	179.0	3.490	0.226	0.903
Increment (cm)	7.4^b^	9.0^ab^	10.8^a^	0.792	0.024	0.859

LCP, low crude protein; MCP, medium crude protein; HCP, high crude protein; SEM, standard error of mean; DMI, dry matter intake; ADG, average daily gain; G:F, gain-to-feed ratio.

a,b Within the same row with different superscripts means significant difference (p<0.05).
